# Association of *Fas* -1377 G/A Polymorphism with Susceptibility to Cancer

**DOI:** 10.1371/journal.pone.0088748

**Published:** 2014-02-18

**Authors:** Peiliang Geng, Jianjun Li, Juanjuan Ou, Ganfeng Xie, Ning Wang, Lisha Xiang, Rina Sa, Chen Liu, Hongtao Li, Houjie Liang

**Affiliations:** Department of Oncology and Southwest Cancer Center, Southwest Hospital, Third Military Medical University, Chongqing, P.R. China; University of North Carolina School of Medicine, United States of America

## Abstract

**Background:**

The relationship between *Fas* -1377 G/A polymorphism and cancer susceptibility has been implicated in accumulating data. However, the data presented inconsistent results. This study was devised to investigate the association of *Fas* -1377 G/A polymorphism and cancer susceptibility in a large number of participants.

**Methods:**

The databases of PubMed, Embase, and Web of Science were searched and a total of 27 case-control studies including 13,355 cases and 16,078 controls were included in this meta-analysis. Pooled odds ratios (ORs) with 95% confidence intervals (CIs) were calculated using the fixed-effects model. Statistical analyses were performed by using Stata software.

**Results:**

The results suggested that *Fas* -1377 G/A polymorphism was overall associated with cancer susceptibility (additive model: OR, 1.16, 95%CI = 1.06–1.27, *P*_heterogeneity_  = 0.381; recessive model: OR, 1.19, 95%CI = 1.10–1.29, *P*_heterogeneity_  = 0.137). In the subgroup analysis by cancer type, significantly increased risk was observed in breast cancer (additive model: OR, 1.24, 95%CI = 1.04–1.58, *P*_heterogeneity_  = 0.614; recessive model: OR, 1.24, 95%CI = 1.02–1.51, *P*_heterogeneity_  = 0.349) and lung cancer (recessive model: OR, 1.25, 95%CI = 1.04–1.49, *P*_heterogeneity_  = 0.090). Similarly, elevated cancer risk associated with *Fas* -1377 G/A polymorphism was revealed in Asians.

**Conclusions:**

The combined results suggest that *Fas* -1377 G/A polymorphism might modulate cancer susceptibility in an Asian-specific manner.

## Introduction

Cancer arises as a result of complex interactions between genetic and environmental factors and has become a major public health problem all over the world [1]–[5]. In recent years, many individual studies have set out to determine whether there is an association between genetic polymorphisms and cancer susceptibility, such as *Fas* -1377 G/A polymorphism and cancer susceptibility. However, these studies showed conflicting results that failed to provide compelling evidence for cancer susceptibility [6]–[9].

Apoptosis is a process of programmed cell death regulated by genes. Inappropriate regulation of apoptosis could lead to a broad range of human disorders including cancer [10]–[13]. *Fas* is a member of the tumor necrosis factor receptor superfamily and regulates apoptotic activities in activated lymphocytes [14]. Located on chromosome 10q24.1, *Fas* is highly polymorphic [15]. A functional polymorphism with a G to A substitution at -1377 position within the *Fas* gene has been extensively explored in the field of cancer. But there is no decisive conclusion of the role of this polymorphism in cancer development [6], [7]. In addition, several studies have been subsequently published since a previous meta-analysis was reported in 2009 [47]. In view of this, we decided to carry out a meta-analysis including 27 eligible studies published to date to systematically and comprehensively estimate the association between *Fas* -1377 G/A polymorphism and susceptibility to cancer.

## Materials and Methods

### Literature Search Strategy

The databases of PubMed, Embase, and Web of Science were searched (the last search was updated in May 2013) to identify all relevant publications on the association between *Fas* -1377 G/A polymorphism and cancer risk. The following search terms and their synonyms were used: “*Fas*”, “1377 G/A” or “CD95” or “rs2234767”, “polymorphism” or “variation”, and “cancer”. We also manually searched the reference lists of all eligible studies and review articles to obtain additional usable data that can be included in the current meta-analysis.

### Inclusion Criteria and Exclusion Criteria

We selected eligible studies according to the following criteria: (1) the study must have a case-control design; (2) the association between *Fas* -1377 G/A polymorphisms and cancer risk must be examined; (3) adequate genotyping data must be contained such that odds ratios (ORs) with 95% confidence intervals (CIs) could be calculated; (4) the study had to be published in English and use human subjects. Exclusion criteria were: (1) insufficient information on the distribution of *Fas* -1377 genotypes; (2) case-only studies; (3) duplicated publications. If a study was subsequently updated, we selected the study with the largest sample size. Two investigators independently reviewed all studies to examine whether they fulfilled the inclusion criteria.

### Data Extraction

Two independent investigators (Peiliang Geng and Jianjun Li) extracted the original data according to the inclusion criteria and exclusion criteria to ensure the accuracy of the retrieved information. The data extracted from each eligible study included the first author's name, year of publication, cancer type, ethnicity, source of controls, method adopted for genotyping, number of cases and controls and genotype frequencies. Disputes were settled by consulting the third person (Houjie Liang).

### Statistical Analysis

Crude ORs with 95% CIs were calculated to evaluate the strength of the association between *Fas* -1377 G/A polymorphism and cancer risk. The pooled ORs were performed for additive model, dominant model and recessive model. Subgroup analysis by cancer type, ethnicity and source of control were also conducted to further assess if the *Fas* -1377 polymorphism was associated with cancer susceptibility in each subgroup. Heterogeneity assumption was evaluated by the chi-square based Q-test and I^2^ statistics [16], [17], *P*>0.05 for the Q test or I^2^<50% suggested a lack of heterogeneity. In this situation, the OR of each study was calculated by the fixed-effects model (the Mantel-Haenszel method) [18]. If *P*<0.05 or I^2^>50%, the random-effects model (the DerSimonian and Laird method) was used [19]. Sensitivity analysis was performed by removing one study at a time to ensure that our findings were not driven by any single study. The evaluation of potential publication bias was performed using the Begg's funnel plots and Egger's test [20]. Hardy-Weinberg equilibrium (HWE) of the control groups was tested by the χ^2^ test for goodness of fitness. All statistical analyses were performed by STATA version 12.0 (Stata Corporation, College Station, TX, USA). A level of *P*<0.05 was accepted as statistically significant.

## Results

### Study Characteristics

**Figure 1:**
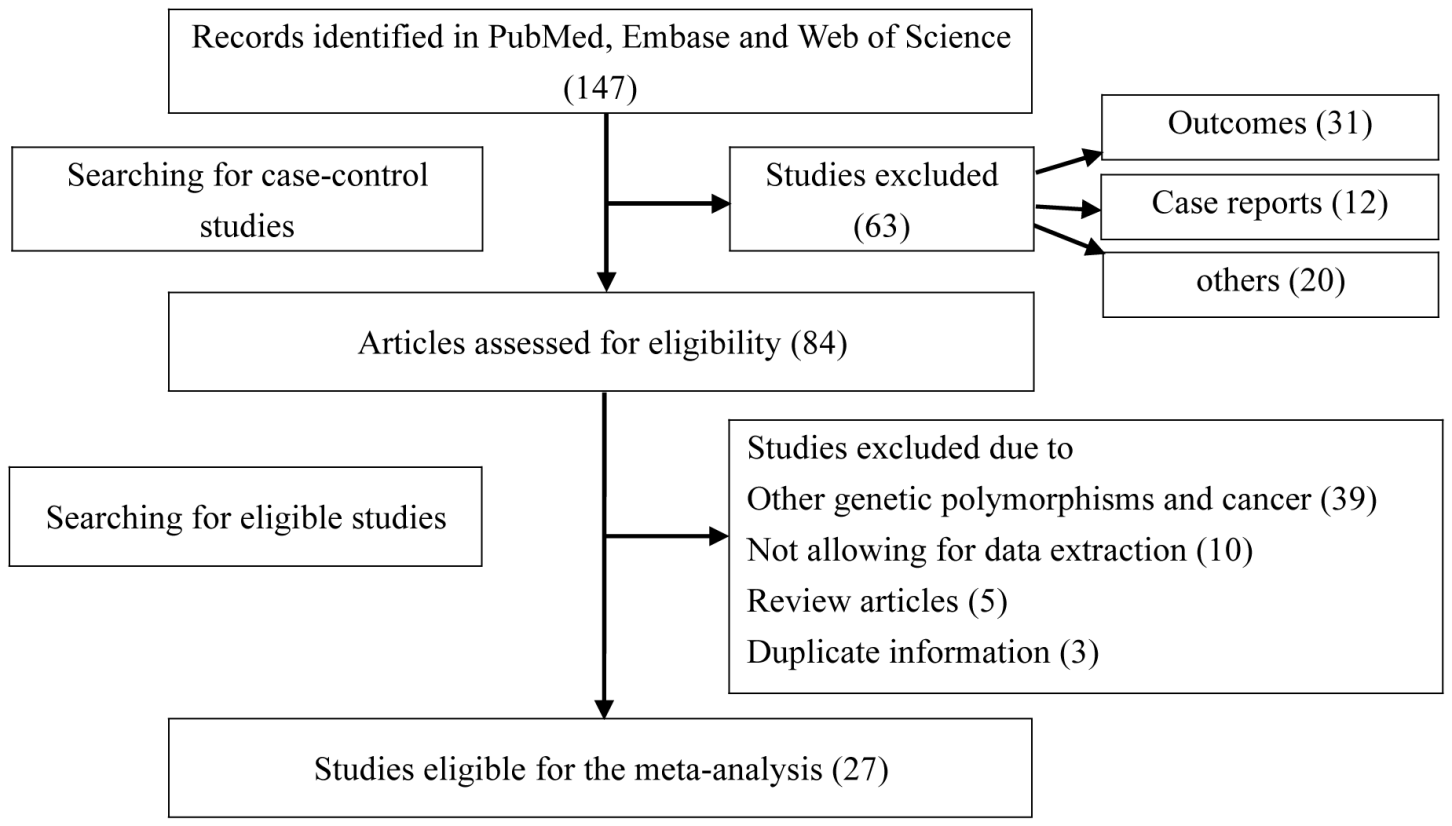
Flow diagram of study identification.

We initially identified 147 potentially relevant studies, of which 27 met the pre-described inclusion criteria and were included in the meta-analysis of the association between Fas -1377G/A polymorphism and cancer risk (Figure 1). Characteristics of all eligible case-control studies for the relationship of *Fas* -1377G/A polymorphism with cancer risk are summarized in Table 1. Of the twenty-seven studies included, an array of cancers including AML [6], [7], breast cancer [21]–[25], cervical cancer [26]–[28], lung cancer [8], [9], [29], [30], gastric cancer [31], [32], melanoma [33], [34], oral cancer [35], [36], and several other cancers [37]–[43] were involved. The subgroup analysis was carried out by cancer type, ethnicity and source of control, respectively. Genotype frequencies were available in all of the 27 studies.

**Table 1:** Main characteristics of the 27 eligible studies.

Authors	Year	Source of control	Ethnicity	Cancer type	Genotyping method	Case						Control						HWE
						Sample size	GG	GA	AA	G	A	Sample size	GG	GA	AA	G	A	
Sibley	2003	Population	European	AML	PCR–RFLP	471	319	136	16	774	168	931	726	186	19	1638	224	0.087
Sun	2004	Population	Asian	Esophageal	PCR–RFLP	588	250	234	104	734	442	648	273	306	69	852	444	0.218
Kripple	2004	Population	European	Breast	TaqMan	499	371	120	8	862	136	497	401	92	4	894	100	0.610
Lai	2005	Hospital	Asian	Cervical	TaqMan	318	127	138	53	392	244	318	99	165	54	3633	273	0.293
Sun	2005	Population	Asian	Cervical	PCR–RFLP	314	144	144	26	432	196	615	282	277	56	841	389	0.304
Zhang	2005	Population	Asian	Lung	PCR–RFLP	1000	413	433	154	1259	741	1270	539	601	130	1679	861	0.046
Li	2006	Hospital	Asian	Bladder	PCR–RFLP	216	66	104	46	236	196	252	81	124	47	286	218	0.970
Park	2006	Hospital	Asian	Lung	PCR–RFLP	582	187	300	95	674	490	582	172	313	97	657	507	0.024
Li	2006	Hospital	European	Melanoma	PCR–RFLP	602	486	107	9	1079	125	603	459	134	10	1052	154	0.951
Zhang	2006	Hospital	European	SCCHN	PCR–RFLP	721	562	142	17	1266	176	1234	957	264	13	2178	290	0.268
Gormas	2007	Population	European	Lung	PCR	94	21	73	0	115	73	50	13	37	0	63	37	>0.05
Zhang	2007	Population	Asian	Breast	PCR–RFLP	840	293	418	129	1004	676	839	345	382	112	1072	606	0.700
Crew	2007	Population	European	Breast	TaqMan	1057	809	225	23	1843	271	1106	847	234	25	1928	284	0.069
Koshkina	2007	Hospital	European	Osteosarcoma	PCR–RFLP	123	99	22	2	220	26	510	400	100	10	900	120	0.210
Zhang	2007	Population	European	Melanoma	PCR–RFLP	229	183	41	5	407	51	351	269	70	12	608	94	0.009
Ter-Minassi	2008	Hospital	European	Lung	TaqMan	2174	1645	492	37	3782	566	1497	1138	336	23	2612	382	0.751
Kang	2008	Population	Asian	Cervical	PCR–RFLP	154	54	69	31	177	131	168	56	82	20	194	142	0.998
Yang	2008	Population	Asian	Pancreatic	PCR–RFLP	397	186	169	42	541	253	907	420	376	111	1216	598	0.062
Zhou	2009	Population	Asian	Gastric	PCR–RFLP	262	124	117	21	365	159	524	225	251	48	701	347	0.062
Cao	2010	Population	Asian	Nasopharyngeal	PCR–RFLP	576	141	264	171	546	606	608	172	303	133	647	569	0.984
Kim	2010	Population	Asian	AML	PCR	592	195	303	94	693	491	858	286	427	145	999	717	0.501
Wang	2010	Population	Asian	Oral	PCR–RFLP	431	146	208	77	500	362	333	115	165	53	395	271	0.628
Zhu	2010	Hospital	Asian	Renal	PCR–RFLP	353	124	173	56	421	285	365	161	161	43	483	247	0.777
Kupcinskas	2011	Hospital	European	Gastric	TaqMan	114	95	18	1	208	20	238	197	40	1	434	42	0.492
Wang	2012	Hospital	Asian	Breast	PCR–RFLP	375	138	171	66	447	303	496	197	246	53	640	352	0.064
Hashemi	2013	Population	Asian	Breast	PCR	134	20	106	8	146	122	152	26	115	11	167	137	>0.05
Karimi	2013	Population	Asian	Oral	PCR–RFLP	139	88	42	9	218	60	126	84	30	12	198	54	0.001

PCR: polymerase chain reaction; PCR-RFLP: PCR-restriction fragment length polymorphism; TaqMan: TaqManSNP; AML: acute myeloid leukemia; SCCHN: squamous cell carcinoma of the head and neck; HWE: Hardy-Weinberg equilibrium.

### Meta-analysis

**Figure 2:**
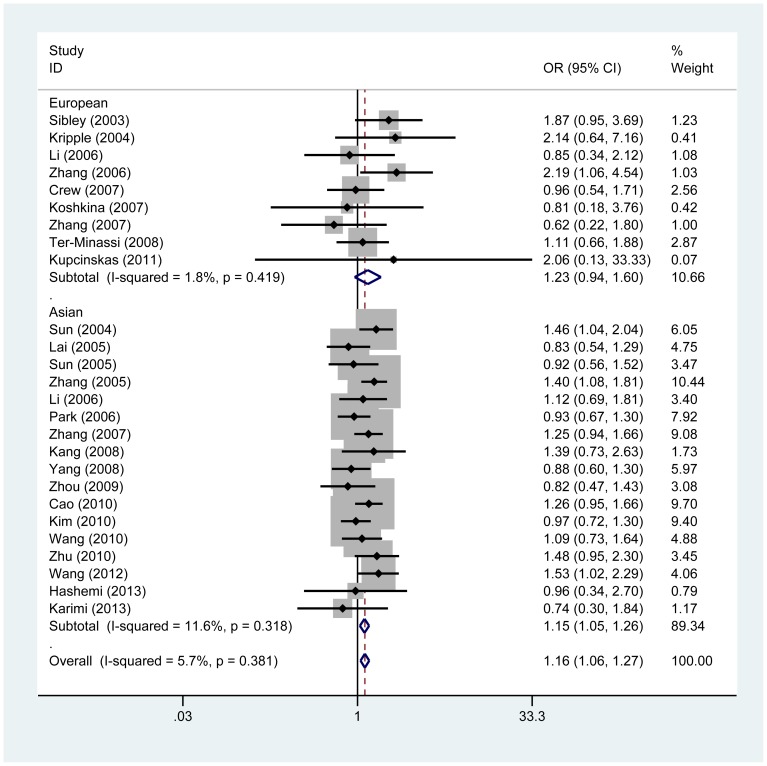
Meta-analysis for the association between Fas -1377 G/A polymorphism and cancer risk by fixed-effects model (additive model; stratified by ethnicity).

**Figure 3:**
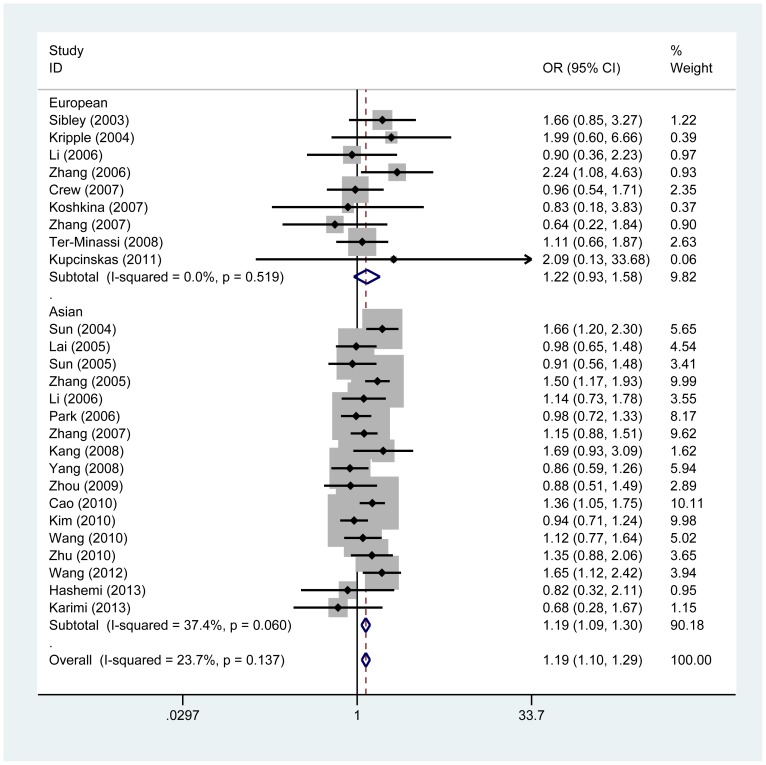
Meta-analysis for the association between Fas -1377 G/A polymorphism and cancer risk by fixed-effects model (recessive model; stratified by ethnicity).

Major results of the meta-analysis are presented in Table 2. No significant between-study heterogeneity was detected across studies and thus we selected the fix-effects model to summarize the ORs. Overall, we found a significant association between *Fas* -1377G/A polymorphism and cancer risk under the additive model (OR, 1.16, 95%CI = 1.06–1.27, *P*_heterogeneity_  = 0.381), but the association was more pronounced under the recessive model (OR, 1.19, 95%CI = 1.10–1.29, *P*_heterogeneity_  = 0.137) (Figure 2, 3). In the subgroup analysis by cancer type, significantly increased risk was observed in breast cancer (additive model: OR, 1.24, 95%CI = 1.04–1.58, *P*_heterogeneity_  = 0.614; recessive model: OR, 1.24, 95%CI = 1.02–1.51, *P*_heterogeneity_  = 0.349) and lung cancer (recessive model: OR, 1.25, 95%CI = 1.04–1.49, *P*_heterogeneity_  = 0.090).

**Table 2:** Main results of the pooled data in the meta-analysis.

	Additive model			Dominant model			Recessive model		
Subtypes	OR (95% CI)	Heterogeneity		OR (95% CI)	Heterogeneity		OR (95% CI)	Heterogeneity	
		P_h_	I^2^ (%)		P_h_	I^2^ (%)		P_h_	I^2^ (%)
Cancer type									
AML	1.07 (0.82, 1.14)	0.080	67.4	1.14 (0.99, 1.30)	0.011	84.6	1.02 (0.79, 1.32)	0.125	57.6
Breast	**1.28 (1.04, 1.58**)	0.614	0	1.08 (0.99, 1.19)	0.602	0	**1.24 (1.02, 1.51)**	0.349	10.0
Cervical	0.96 (0.72, 1.29)	0.432	0	0.96 (0.83, 1.12)	0.590	0	1.08 (0.82, 1.42)	0.245	28.9
Lung	1.19 (0.98, 1.43)	0.163	45.0	1.01 (0.92, 1.10)	0.960	0	**1.25 (1.04, 1.49)**	0.090	58.4
Melanoma	0.74 (0.37, 1.47)	0.659	0	0.82 (0.66, 1.03)	0.795	0	0.77 (0.39, 1.53)	0.627	0
Gastric	0.85 (0.50, 1.46)	0.526	0	0.93 (0.74, 1.17)	0.885	0	0.90 (0.53, 1.52)	0.547	0
Oral	1.03 (0.71, 1.48)	0.444	0	1.03 (0.84, 1.26)	0.749	0	1.04 (0.74, 1.47)	0.313	1.9
Other	1.26 (1.08, 1.47)	0.301	16.9	1.02 (0.94, 1.11)	0.940	0	1.31 (1.13, 1.52)	0.139	38.0
Ethnicity									
European	1.23 (0.94, 1.60)	0.419	1.8	1.04 (0.96, 1.13)	0.069	43.4	1.22 (0.93, 1.58)	0.519	0
Asian	**1.15 (1.05, 1.26)**	0.318	11.6	1.02 (0.97, 1.07)	0.994	0	**1.19 (1.09, 1.30)**	0.060	37.4
Source of control									
Population	**1.16 (1.05, 1.29)**	0.383	6.1	1.05 (0.99, 1.10)	0.587	0	**1.19 (1.08, 1.32)**	0.073	36.4
Hospital	1.15 (0.97, 1.35)	0.311	14.3	0.99 (0.92, 1.06)	0.783	0	**1.19 (1.02, 1.39)**	0.419	2.2
Total	**1.16 (1.06, 1.27)**	0.381	5.7	1.02 (0.98, 1.07)	0.722	0	**1.19 (1.10, 1.29)**	0.137	23.7
Total*	1.16 (1.05, 1.28)	0.484	0	1.03 (0.98, 1.08)	0.583	0	1.19 (1.08, 1.30)	0.249	15.8

AML: acute myeloid leukemia; P_h_: p-value of heterogeneity test; CI: confidence interval; OR: odds ratio;

^*^meta-analysis results after removing the studies deviating from Hardy-Weinberg equilibrium (HWE).

Subgroup analysis by ethnicity also provided evidence for an association in Asian populations (additive model: OR, 1.15, 95%CI = 1.05–1.26, *P*_heterogeneity_  = 0.318; recessive model: OR, 1.19, 95%CI = 1.09–1.30, *P*_heterogeneity_  = 0.060), but not in European populations. In the succeeding analysis by source of control, an elevated cancer risk was observed in both population-based and hospital-based studies (Table 2).

### Sensitivity Analysis

We performed a leave-one-out sensitivity analysis by omitting one study at a time to assess the stability of the combined results. The results suggested that our findings were not substantially affected by any single study (data not shown).

### Publication Bias

**Figure 4:**
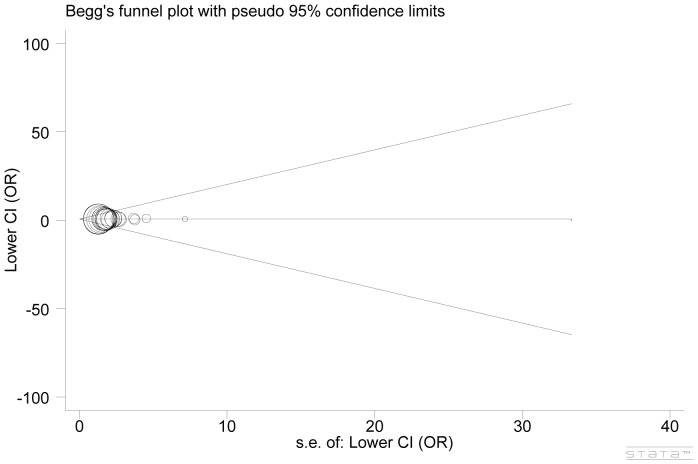
Publication bias test for all included studies (additive model).

Begg's funnel plot and Egger's test were performed to detect publication bias. No statistically significant evidence of publication bias was revealed (Begg's test: *P* = 0.826; Egger's test: *P* = 0.721, additive model) (Figure 4).

## Discussion

The human *Fas* gene mapped on chromosome 10q24.1 consists of nine exons and eight introns [15]. -1377 G/A polymorphism, located in the promoter region of the *Fas* gene, has been investigated in a variety of previous studies looking at cancer risk [8], [21], [22], [26]. However, these findings remain controversial rather than conclusive. This might be attributed to the different ethnicities, distinct study design, and sample inadequacy in each of the published studies. But meta-analysis could avoid the shortcomings and convincingly estimate the genetic association through including all relevant studies.

In our meta-analysis, we observed *Fas* -1377G/A polymorphism was overall associated with cancer susceptibility under the additive model and the recessive model. Several published meta-analyses observed the same finding that *Fas* -1377 G/A polymorphism was associated with cancer risk as well as some common diseases, such as autoimmune rheumatic diseases, systemic lupus erythematosus [44]–[47]. The detection power of the four meta-analyses, however, may be limited largely because of sample insufficiency: 4 publications (996 cases and 1,160 controls) were included by Lu et al. [44], 5 (615 cases and 622 controls) by Lee et al. [45], 3 (444 cases and 442 controls) by Xiang et al. [46] and 17 (10,564 cases and 12,075 controls) by Qiu et al [47]. Our meta-analysis nevertheless summarized data from 27 studies composed of 13,355 cases and 16,078 controls. It should be noted that study size is obviously important to know the proportion of false positive findings of meta-analysis. Therefore, the relatively larger sample may assure the statistical power of our study. Deviation from HWE was observed in several studies, which may result from misclassification of genotypes, because multiple genotyping methods were used across studies. When we reanalyzed the studies without departure form HWE, the general results were not significantly altered, suggesting our findings are robust and convincing.

Apart from the comparison among all subjects, we also performed stratification analysis by cancer type. We found that *Fas* -1377 G/A polymorphism increased the risk of some cancers, such as breast cancer and lung cancer. Our findings were consistent with those revealed in the previous studies [6], [9], [21], [26], but contradictory discoveries that there was no association between *Fas* -1377 G/A polymorphism and lung cancer were also suggested in two studies [7], [8]. The underlying etiology mechanisms differ substantially across cancers, and the role of *Fas* -1377 G/A polymorphism in various caners requires to be identified by future larger studies.

In addition, in the subgroup analysis by ethnicity, *Fas* -1377 G/A polymorphism was found to increase cancer risk in Asian populations under several genetic models, such as the recessive model and the additive model. However, this association was obtained in European populations. There is obvious disparity in genotype frequencies between the two ethnic groups (GA: 21.3% vs 47.7%; AA: 1.5% vs 13.2%). It is known that different genetic background donates a series of differences between ethnic groups, for instance, frequency of exposure to cancer-causing agents and diverse lifestyles, which are important components in the process of cancer progression.

In the final subgroup analysis by control source, we observed significant association in both population-based and hospital-based studies. However, investigators demonstrated a different discovery of significantly increased cancer risk associated with *Fas* -1377 AA genotype among studies based on population-based controls, but not among studies of hospital-based controls [47]. Control subjects in some hospital-based studies may be poorly-defined reference populations and failed to well represent the general population, leading to some biases in the analysis, but the relatively small sample may be responsible for a large part of the inconsistency.

Some limitations in our meta-analysis need to be addressed. To begin with, in the subgroup analysis by cancer type, significant association was not observed in several cancers, such as gastric cancer, melanoma cancer and oral cancer. *Fas* -1377 G/A polymorphism and these cancers may be positively correlated, which may be masked due to the small sample size in this study. Furthermore, there existed heterogeneity between studies. The reason might be attributable to the different genetic backgrounds of the subjects and study design in each of the included studies. Finally, this meta-analysis was carried out among Asian and European populations, thus the results can not be applicable in other ethnicities.

In summary, the meta-analysis provided evidence that *Fas* -1377 G/A polymorphism might be associated with an increased cancer risk. Significant association was also found in subgroup analyses by cancer type, ethnicity and source of control. In future, studies with a larger sample size and multiple ethnic groups are required to further validate the relationship between *Fas* -1377 G/A polymorphism and cancer susceptibility.
